# Visual and Quantitative Evaluation of Low-Concentration Bismuth in Dual-Contrast Imaging of Iodine and Bismuth Using Clinical Photon-Counting CT

**DOI:** 10.3390/s24113567

**Published:** 2024-06-01

**Authors:** Afrouz Ataei, Vasantha Vasan, Todd C. Soesbe, Cecelia C. Brewington, Zhongxing Zhou, Lifeng Yu, Kristina A. Hallam, Liqiang Ren

**Affiliations:** 1 Department of Radiology, UT Southwestern Medical Center, Dallas, TX 75390, USA; 2 Department of Radiology, Mayo Clinic, Rochester, MN 55905, USA; 3 Siemens Medical Solutions USA Inc., Malvern, PA 19355, USA

**Keywords:** photon-counting CT, dual-contrast imaging, iodine and bismuth

## Abstract

Simultaneous dual-contrast imaging of iodine and bismuth has shown promise in prior phantom and animal studies utilizing spectral CT. However, it is noted that in previous studies, Pepto-Bismol has frequently been employed as the source of bismuth, exceeding the recommended levels for human subjects. This investigation sought to assess the feasibility of visually differentiating and precisely quantifying low-concentration bismuth using clinical dual-source photon-counting CT (PCCT) in a scenario involving both iodinated and bismuth-based contrast materials. Four bismuth samples (0.6, 1.3, 2.5, and 5.1 mg/mL) were prepared using Pepto-Bismol, alongside three iodine rods (1, 2, and 5 mg/mL), inserted into multi-energy CT phantoms with three different sizes, and scanned on a PCCT system at three tube potentials (120, 140, and Sn140 kV). A generic image-based three-material decomposition method generated iodine and bismuth maps, with mean mass concentrations and noise levels measured. The root-mean-square errors for iodine and bismuth determined the optimal tube potential. The tube potential of 140 kV demonstrated optimal quantification performance when both iodine and bismuth were considered. Distinct differentiation of iodine rods with all three concentrations and bismuth samples with mass concentrations ≥ 1.3 mg/mL was observed across all phantom sizes at the optimal kV setting.

## 1. Introduction

In recent years, there has been growing interest in the field of diagnostic radiology towards the separation of two or more contrast materials (i.e., dual- or multi-contrast imaging) using a single scan of spectral CT [1,2,3,4,5,6]. Among various combinations of contrast materials, dual-contrast imaging of iodine and bismuth has shown promise in small-bowel imaging, enabling separation of the iodine-enhanced bowel wall and bismuth-enhanced bowel lumen [7,8]. This innovative imaging approach holds great potential for improved detection of bowel pathology, especially in small-bowel examinations. For instance, in cases of suspected small-bowel wall pathology such as Crohn’s disease or small-bowel tumors, dual-contrast imaging allows for improved assessment of the mucosal/luminal aspect of the bowel wall. This integrated information provides a more comprehensive understanding of pathology, aiding in accurate diagnosis [7,9].

In animal studies, Pepto-Bismol, containing bismuth subsalicylate, has commonly been used as the oral contrast material [7,8,10]. However, the dosage administered in these studies often exceeds the recommended usage, which at regular strength is one dose (30 mL) every half hour or two doses (60 mL) every hour, as required, with a maximum limit of eight doses (240 mL) within a 24 h period. None of the existing animal studies, however, followed the recommended dose usage. For example, 120 to 180 mL bismuth subsalicylate was orally administered in two different rabbit abdominopelvic models as a single bolus in addition to intravenous iodine injection and scanned on dual-energy CT (DECT) [8,11,12]. Although the radiologists’ diagnostic confidence was increased, the amount of administered intraluminal bismuth contrast medium was about 4 to 6 doses, much higher than the recommended dose usage, given the size differences between rabbits and humans. Similar dose levels (180 mL bismuth subsalicylate) were used in two swine studies to differentiate the iodine-enhanced small-bowel wall and bismuth-filled small-bowel loop on an iodine map and bismuth map after spectral CT scans and material decomposition [10,13]. It is reasonable to assume that the use of a large amount of contrast materials may improve the signal detectability and quantification accuracy; however, this poses great challenges to the clinical translation of this dual-contrast imaging technique to human subjects.

To bridge the technical gap preventing potential clinical translations, this study aims to assess the feasibility of visually differentiating and accurately quantifying low-concentration bismuth in a dual-contrast imaging task involving both iodinated and bismuth-based contrast materials on a clinical dual-source photon-counting CT (PCCT) and using a custom three-material decomposition method [14].

## 2. Materials and Methods

### 2.1. Bismuth Sample Preparation and Phantom Design

Four bismuth samples were prepared by progressively diluting bismuth subsalicylate (Pepto-Bismol, Procter & Gamble, Mason, OH, USA; original bismuth concentration: 10.1 mg/mL) to achieve the following concentration levels: 5.1, 2.5, 1.3, and 0.6 mg/mL. The low-concentration bismuth samples were prepared strictly following the recommended usage dosage of bismuth subsalicylate (one dose (30 mL) every half hour or two doses (60 mL) every hour) such that the concentration of 0.6 mg/mL was calculated by mixing one dose of Pepto-Bismol with 500 mL water or two doses with 1000 mL water. The second-lowest concentration of 1.3 mg/mL was calculated by mixing two doses of Pepto-Bismol with 500 mL water. To simulate scenarios where patients have adequate time (e.g., 2 to 4 h) before a CT scan, allowing the administration of multiple doses of Pepto-Bismol, higher bismuth concentration levels such as 2.5 and 5.1 mg/mL were also prepared.

**Figure 1:**
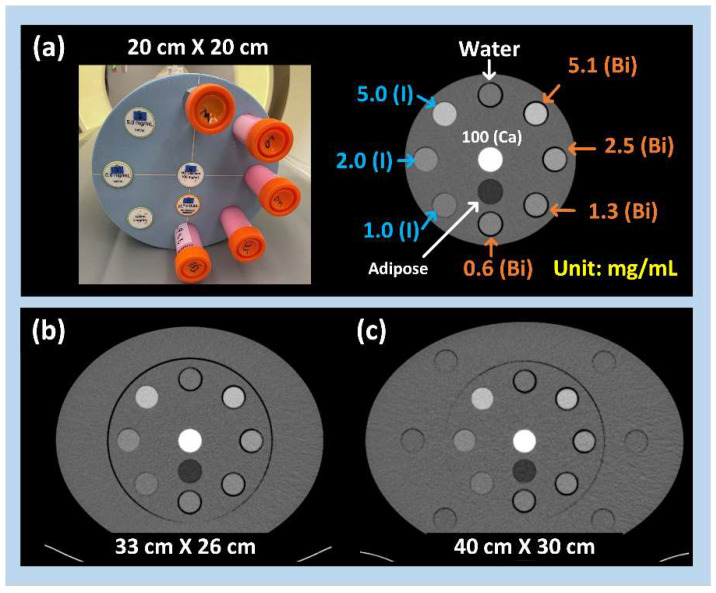
Four bismuth samples, three iodine rods, and three other materials (pure water, calcium, and adipose) were inserted into multi-energy CT phantoms with three sizes, (**a**) small: 20 cm × 20 cm, (**b**) medium: 33 cm × 26 cm, and (**c**) large: 40 cm × 30 cm. Layout and concentrations of bismuth samples, iodine rods, and three other materials were labeled on an example CT image acquired from the small phantom.

The four prepared bismuth samples; three solid iodine rods (Sun Nuclear, A Mirion Medial Company, Melbourne, FL, USA) at concentrations of 1, 2, and 5 mg/mL; and a pure water sample were inserted into a small multi-energy CT phantom (20 cm × 20 cm). The phantom could be accommodated within two body rings (33 cm × 26 cm; 40 cm × 30 cm), allowing for the replication of patients with medium and large sizes (Sun Nuclear, A Mirion Medial Company, Melbourne, FL, USA). Two other rods (Calcium at 100 mg/mL and General Adipose; Sun Nuclear, A Mirion Medial Company, Melbourne, FL, USA) were also imaged to evaluate their spectral performance on iodine and bismuth maps. For further clarity, the layout and concentration levels of these samples and rods within the multi-energy CT phantoms are depicted and labeled in Figure 1.

### 2.2. PCCT Data Acquisition and Reconstruction

A clinical PCCT system (NAEOTOM Alpha VA50_sp1, Siemens Healthineers GmbH, Forchheim, Germany) was employed to scan all three phantoms. All PCCT scans were conducted in multi-energy mode using three tube potentials: 120, 140, and Sn140 kV (Sn: 0.4 mm tin filter). The 120 kV and 140 kV settings were chosen from QuantumPlus mode, and Sn140 kV was selected from QuantumSn mode. Each of these selected tube potentials had two energy thresholds. The low-energy threshold was fixed to eliminate electronic noise, while the high-energy varied depending on the selected tube potentials. The radiation doses indicated by volume CT dose index (CTDIvol) were determined for each phantom size at 120 kV with automatic exposure control (AEC) enabled with an image quality (IQ) level set to 145. The determined CTDIvol values were 3, 8, and 12 mGy for the small, medium, and large phantoms, respectively, matched across all scans with three kVs. For each scan, low- and high-energy images (referred to as “L/H” on the scanner console) were reconstructed with a medium-smooth quantitative kernel (Qr40) using quantum iterative reconstruction algorithm at a strength level of 3 (QIR-3). All images reconstructed directly from the scanner console had slice thickness of 2.0 mm, which is the maximum allowed thickness, and then were reformatted to 3.0 mm using the “parallel range” function in syngo.via (VB60A, Siemens Healthineers GmbH, Forchheim, Germany). The selection of 3 mm thickness was based on our clinical protocol for CT enterography. Table 1 summarizes essential scan and reconstruction parameters.

### 2.3. Material Decomposition

Three-material decomposition was performed using a generic image-based algorithm to separate and quantify each basis material (iodine, bismuth, and water) using the low- and high-energy images from PCCT. The material decomposition process was mathematically performed pixel by pixel and is described in Equation (1) [15]:

(1) $$\left\{\begin{array}{c} CT\left(E_{L}\right)=M_{Iod}\left(E_{L}\right)\cdot \rho_{Iod}+M_{Bis}\left(E_{L}\right)\cdot \rho_{Bis}\\ CT\left(E_{H}\right)=M_{Iod}\left(E_{H}\right)\cdot \rho_{Iod}+M_{Bis}\left(E_{H}\right)\cdot \rho_{Bis} \end{array}\;\;\;\Leftrightarrow \;\;\vec{CT}=M\vec{\rho }\right.$$

where $\vec{CT}=\left[\begin{array}{c} CT\left(E_{L}\right)\\ CT\left(E_{H}\right) \end{array}\right]$ represents the vector of CT numbers measured on the low-energy ($E_{L}$) and high-energy ($E_{H}$) images from PCCT; $\vec{\rho }=\left[\begin{array}{c} \rho_{Iod}\\ \rho_{Bis} \end{array}\right]$ denotes the vector of mass concentrations to be calculated for iodine and bismuth in the unit of mg/mL; and $M=\left[\begin{array}{cc} M_{Iod}\left(E_{L}\right) & M_{Bis}\left(E_{L}\right)\\ M_{Iod}\left(E_{H}\right) & M_{Bis}\left(E_{H}\right) \end{array}\right]$ describes the coefficient matrix that can be calibrated and determined beforehand by separate high-dose scans of iodine and bismuth samples with known mass concentrations using the same PCCT techniques.

As previously reported [15,16], in the image-based three-material decomposition process described above, CT numbers could be utilized directly instead of linear attenuation coefficients. By doing so, this method inherently incorporates the assumption of volume conservation, wherein the total volume of the mixture equals the sum of the volumes of each basis material, thereby adding an additional physical constraint. Thus, the water mass concentration ($\rho_{Water}$) at each pixel can be solved in Equation (2):

(2) $$\rho_{Water}=\rho_{Water,0}\left(1-\frac{\rho_{Iod}}{\rho_{Iod,0}}-\frac{\rho_{Bis}}{\rho_{Bis,0}}\right)$$

where $\rho_{Water,0}$, $\rho_{Iod,0}$, and $\rho_{Bis,0}$ are the mass densities of water, iodine, and bismuth in their pure forms. After solving the mass concentrations for each base material at the pixel level, iodine, bismuth, and water maps can be generated for qualitative evaluations and quantitative measurements.

### 2.4. Qualitative and Quantitative Analysis

Careful evaluations were conducted on all low- and high-energy images, as well as iodine, bismuth, and water maps derived from three different phantom sizes and tube potentials, to visually assess the contrast material signals, noises, and image artifacts. A single circular region of interest (ROI) was designated for each sample to quantify the mean mass concentrations across all material maps. For each phantom size and tube potential option, the ranges of bias values ($\rho_{measured}-\rho_{nominal}$) were calculated and reported for each contrast material. Within the same ROIs, the noise levels (standard deviation) were also calculated and recorded. Based on the measured mean mass concentrations and standard deviation values, root-mean-square error (RMSE) was calculated for each iodine or bismuth sample. The overall noise levels and RMSE values for iodine (${Noise}_{Iod}$ and ${RMSE}_{Iod}$) and bismuth (${Noise}_{Bis}$ and ${RMSE}_{Bis}$) quantifications were calculated as

(3) $${Noise}_{M}=\sqrt{\frac{1}{N}\sum_{i=1}^{N}{Noise}_{i}^{2}}$$

(4) $${RMSE}_{M}=\sqrt{\frac{1}{N}\sum_{i=1}^{N}{RMSE}_{i}^{2}}$$

where M denotes the contrast material of iodine or bismuth, N is the number of samples, and ${Noise}_{i}$ and ${RMSE}_{i}$ represent the noise level and RMSE for the ith sample. The tube potential corresponding to the lowest overall RMSE values was regarded to be optimal. Since the lowest ${RMSE}_{Iod}$ and ${RMSE}_{Bis}$ may not be acquired at the same tube potential, a joint RMSE defined as ${RMSE}_{joint}=\sqrt{{{RMSE}_{Iod}}^{2}+{{RMSE}_{Bis}}^{2}}$ was used to determine a single optimal tube potential. Paired sample *t*-test was performed to compare the joint RMSE values for each phantom size, and p<0.05 was regarded as statistically different. Compared to other methods such as mean absolute error or signal-to-noise ratio, the RMSE values were calculated incorporating both image noise and quantification bias to comprehensively evaluate the overall quantification performance of each basis material after material decomposition [17,18]. In addition, contrast-to-noise ratios (CNRs) were calculated as $CNR=(\rho_{sample}-\rho_{background})/{SD}_{background}$ for three low-concentration materials (1.0 mg/mL iodine and 0.6 as well as 1.3 mg/mL bismuth) and compared across all tube potentials and phantom sizes.

## 3. Results

### 3.1. Visual and Qualitative Evaluations

#### 3.1.1. Iodine and Bismuth Separations

**Figure 2:**
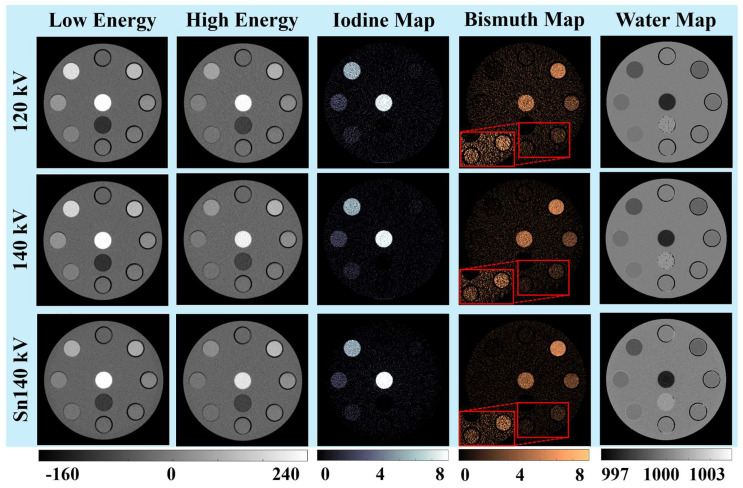
(First–second columns) low- and high-energy PCCT images (window/center: 400/40 HU); (third-fifth columns) iodine (window/center: 8/4 mg/mL), bismuth (overall window/center: 8/4 mg/mL; inset window/center: 3/1.5 mg/mL), and water (window/center: 10/1000 mg/mL) maps; images and maps were acquired for the small phantom.

**Figure 3:**
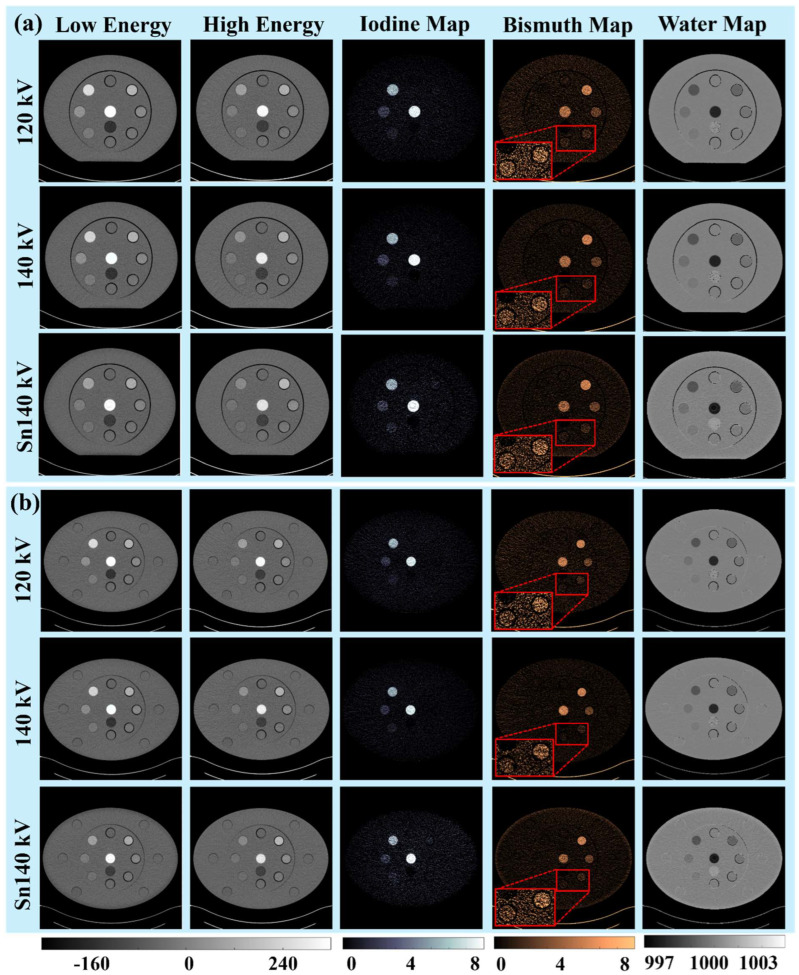
(First–second columns) low- and high-energy PCCT images (window/center: 400/40 HU); (third-fifth columns) iodine (window/center: 8/4 mg/mL), bismuth (overall window/center: 8/4 mg/mL; inset window/center: 3/1.5 mg/mL), and water (window/center: 10/1000 mg/mL) maps; all images and maps acquired for (**a**) medium and (**b**) large phantoms.

Figure 2 and Figure 3 illustrate the PCCT low-energy (first column) and high-energy (second column) images of three phantoms acquired at three tube potentials: 120 kV (first row), 140 kV (second row), and Sn140 kV (third row). The CT numbers of the three iodine rods decreased when the tube potential was increased and the additional tin filter was added, while the effective X-ray beam energy increased from low-energy to high-energy and moved away from the iodine K-edge energy (33.2 keV). Conversely, the CT numbers of four bismuth samples remained stable due to the significantly higher K-edge energy of bismuth (90.5 keV). Despite the distinct energy dependencies of CT numbers on iodine and bismuth, the original PCCT images alone did not readily distinguish between these two materials. However, the subsequent material decomposition and generation of iodine (third column) and bismuth (fourth column) maps successfully identified and separated the contrast materials across all tube potentials and phantom sizes. Additionally, water maps were derived and displayed (fifth column) after removing the contributions of the two contrast materials. Note that the calcium rod showed up on both the iodine map and bismuth map and was completely removed from the water map, whereas the adipose rod was only displayed on the water map.

#### 3.1.2. Signals and Noises

Consistent contrast material signals were observed across all three tube potentials and phantom sizes for iodine samples on the iodine maps and bismuth samples on the bismuth maps. However, there were variations in the overall noise levels. Visually, the lowest noise level was achieved at 140 kV for the iodine map and 140 kV or Sn140 kV for the bismuth map, depending on the phantom sizes. As the phantom sizes increased from small to medium and large, the overall noise levels in all material maps increased accordingly.

#### 3.1.3. Low-Concentration Materials

The iodine maps exhibited a clear identification of the lowest iodine concentration (1 mg/mL), which was more visually distinct in the smaller phantoms. Conversely, discerning the lowest bismuth concentrations (0.6 and 1.3 mg/mL) was less straightforward, requiring a narrower window width for enhanced clarity. Therefore, these two low-concentration bismuth samples were zoomed in on and displayed in a [0, 3 mg/mL] window. Similar to the iodine maps, the lowest concentration of bismuth was also more visibly discernable in the small phantom compared to that in the medium and large phantoms. Despite the elevated noise with increased phantom sizes, the 1.3 mg/mL bismuth sample remained discernible, while the 0.6 mg/mL one was challenging.

### 3.2. Quantitative Evaluations

#### 3.2.1. Quantitative Measurements and Optimal PCCT Tube Potential

On all the iodine and bismuth maps derived from the three tube potentials and three phantom sizes, mean mass concentration (±standard deviation) values were measured and are summarized in Table 2. The range of bias values (measured values–nominal values) were [−0.56, 0.49 mg/mL] for iodine quantifications and [−0.44, 0.29 mg/mL] for bismuth quantifications across all contrast material concentrations, tube potentials, and phantom sizes.

**Figure 4:**
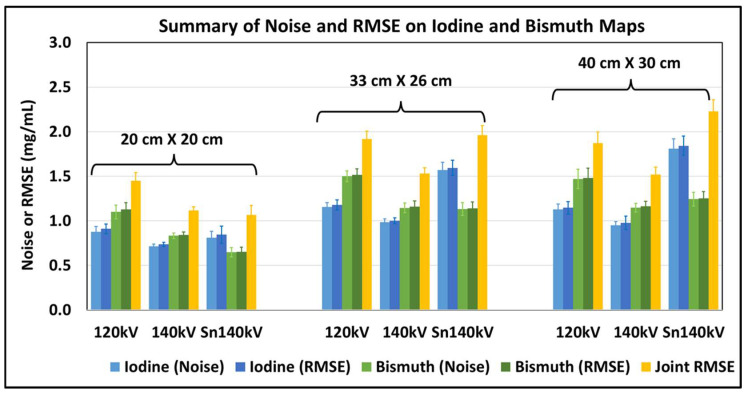
Summary of noise and RMSE values (unit: mg/mL) on iodine and bismuth maps across three tube potentials (120, 140, and Sn140 kV) and three phantom sizes (small, medium, and large).

Noise levels and RMSE values computed on the iodine and bismuth maps across all the contrast material concentrations at three kV settings and three different phantom sizes are illustrated in Figure 4. The overall trend of noise levels was the same as that of the RMSE values, and the small difference between them (range: [0.01, 0.03 mg/mL]) indicated that the quantification bias values were very small. Being consistent with the visual observations, the lowest noise level was achieved at 140 kV for the iodine map for all the phantom sizes and 140 kV or Sn140 kV for the bismuth map depending on the phantom size. Similarly, the lowest iodine RMSE values were achieved at 140 kV for all phantoms, namely, 0.74, 1.00, and 0.98 mg/mL for the small, medium, and large phantoms, respectively. The lowest bismuth RMSE values, however, were dependent on the phantom size and attained at either 140 kV or Sn140 kV, with 0.65, 1.14, and 1.16 mg/mL for the small, medium, and large phantom sizes, respectively. As also shown in Figure 4, the calculated joint RMSE values suggested a tube potential of 140 kV or Sn140 kV (p=0.365) to be optimal for both iodine and bismuth with the small phantom and 140 kV to be optimal for medium and large phantoms (p<0.001 compared to 120 kV or Sn140 kV). Considering all phantom sizes, the tube potential of 140 kV is deemed as the optimal selection.

#### 3.2.2. Low-Concentration Materials

**Figure 5:**
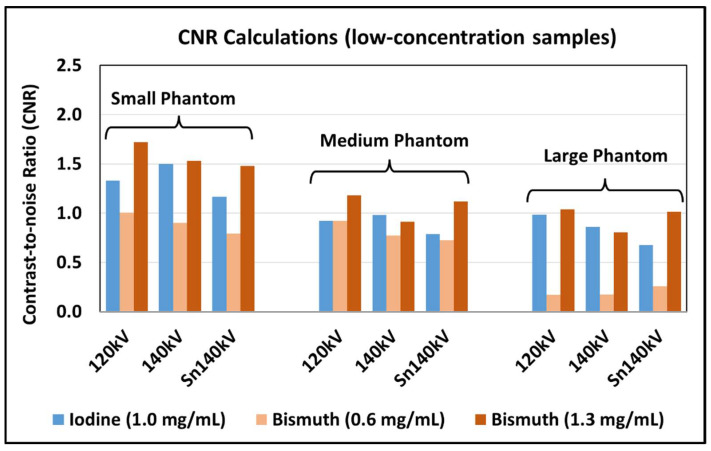
Contrast-to-noise ratios (CNRs) calculated for three low-concentration samples (1.0 mg/mL iodine, 0.6 mg/mL bismuth, and 1.3 mg/mL bismuth) across three tube potentials (120, 140, and Sn140 kV) and three phantom sizes.

The CNR values calculated for three low-concentration materials, namely, 1.0 mg/mL iodine, 0.6 mg/mL bismuth, and 1.3 mg/mL bismuth, are summarized in Figure 5. The 0.6 mg/mL bismuth had CNR values consistently smaller than 1.0 mg/mL iodine and 1.3 mg/mL bismuth across all tube potentials and phantom sizes and therefore was difficult to visualize. The CNR calculations were consistent with the visual observations.

## 4. Discussion

In recent years, several phantom studies have explored the feasibility of the simultaneous imaging of intravenously injected iodinated contrast material and orally administered bismuth-based contrast materials for small-bowel imaging [10,11]. Additionally, a few feasibility animal studies have demonstrated the technical viability of this dual-contrast imaging task using spectral CT [10,13]. Despite these promising findings, successful translation from phantom and animal models to human subjects is hindered by various factors, including the excessive number of bismuth contrast doses used in reported studies for faithful quantification by spectral CT and material decomposition. The results in this study showed that bismuth samples with a mass concentration as low as 1.3 mg/mL can be reliably discernable on decomposed bismuth maps derived using a custom three-material decomposition method based on PCCT images. Detection of the 0.6 mg/mL bismuth sample was challenging as it was very close to the limit of detection (~0.5 mg/mL) for bismuth by CT [19].

Consistent with previous studies, bismuth subsalicylate from Pepto-Bismol served as the oral contrast material in our investigation [7,8,11]. The lowest bismuth concentrations were established at 0.6 and 1.3 mg/mL, achieved by diluting one or two doses of Pepto-Bismol in 500 mL of water. Higher concentration levels, exceeding 1.3 mg/mL, could be attained by administering multiple doses within a specified timeframe (every 30 min within two to four hours), potentially and theoretically reaching concentrations of up to 2.5 and 5.0 mg/mL. These higher bismuth contrast dose levels, however, may not be clinically relevant due to peristalsis in the intestine. While the higher doses may not all be in the small bowel, they are likely to accumulate in the colon and have the potential to increase the identification of colon cancer. Nonetheless, this phantom study marks the first systematic assessment of the imaging performance of low-concentration bismuth samples across multiple phantom sizes, utilizing PCCT scans at various tube potentials.

While phantom studies investigating simultaneous iodine and bismuth imaging have been conducted on multiple dual- or multi-energy CT scanner platforms, such studies have not been performed on the clinical PCCT systems, particularly when involving low-concentration bismuth samples. Thus, it is imperative to ascertain the optimal tube potential for this imaging task to minimize quantification bias, reduce image noise, and achieve the lowest RMSE values. The tube potential of 140 kV was identified as the optimal setting for iodine quantification, whereas 140 kV or Sn140 kV was identified for bismuth quantification. The preference for a tube potential of 140 kV arises from the fact that the K-edge energy of iodine (33.2 keV) is typically too low for X-ray spectra in most CT systems, requiring better spectral separation between the low- and high-energy. Conversely, bismuth’s K-edge energy (90.5 keV) is higher and allows it to be captured by all three X-ray spectra used in the current study. Therefore, a tube potential with more photons allocated near the K-edge energy, such as 140 kV or Sn140 kV, is preferred for bismuth imaging. Since only one tube potential can be applied during an actual CT scan, a joint analysis of RMSE values combining two contrast materials was conducted to determine the single tube potential. The analysis identified 140 kV as the optimal tube potential for this imaging task performed on the clinical PCCT for all three phantom sizes.

The differentiation of both iodine and bismuth in the intestinal lumen is highly relevant in the clinical context, particularly for enabling enteral contrasting in cases of unclear abdominal conditions where intestinal bleeding is a possibility. Currently, enteral contrast administration must be avoided in such scenarios because it impedes the diagnosis of bleeding, thereby reducing diagnostic accuracy for passage problems. This clinical scenario may be simulated by preparing and imaging mixtures comprising both contrast materials. Previous studies have demonstrated successful separation and quantification of iodine and bismuth when they were mixed, and the clinical PCCT is expected to achieve a similar material decomposition performance [10,15].

This study has several limitations. Firstly, all bismuth samples were prepared by mixing it with water, which may yield a different material decomposition performance compared to mixing Pepto-Bismol with other types of neutral oral contrast materials. To enhance clinical applicability, future work may require modified material decomposition methods that account for variations in background materials other than water. Secondly, the energy thresholds and number of energy bins were fixed for each kV, with no provision for user adjustment for K-edge imaging [14]. Enhanced material decomposition at 140 kV may be achievable by increasing the high threshold energy to approach the K-edge energy of bismuth. Future research should explore and investigate flexible energy settings to enhance material decomposition accuracy. Thirdly, even with the optimal tube potential, the noise levels in the decomposed contrast maps were still quite high, preventing confident visualizations of the lowest concentration level (i.e., 0.6 mg/mL). To enhance the spatial orientation and better utilize iodine and bismuth maps, they may be overlaid with the native CT images to improve the visibility of material separations. To faithfully detect the bismuth signals with similar concentration levels encountered in human subjects, advanced material decomposition methods or noise reductions are needed. Material decomposition involving iodine and bismuth may be also improved by operating the two X-ray tubes at different tube potentials, as demonstrated in a previous study with a PCCT research prototype for multi-contrast imaging [17]. This configuration (i.e., quantum-peak mode), however, is not available in the clinical PCCT system software version VA50_sp1 used for this study and will be investigated for the dual-contrast imaging of iodine and bismuth in future studies [20]. Lastly, this study does not take the intraluminal mixing of bismuth with bowel content/fluid into account that would further reduce the bismuth concentration in clinical practice.

## 5. Conclusions

In conclusion, our study contributes to the ongoing exploration of simultaneous imaging of iodinated contrast material and bismuth-based contrast material for small-bowel imaging, aiming to bridge the gap between current preclinical research studies and potential clinical applications. By evaluating the visual and quantitative performance of low-concentration bismuth samples in various phantom sizes using a clinical PCCT, we attempted to address the challenge of excessive contrast doses reported in previous studies, offering insights into the potential translation of this imaging approach to human subjects. Overall, our study represents a significant step forward in the development of dual-contrast imaging techniques for small-bowel imaging. Further refinement of imaging protocols and material decomposition methods holds promises for improving the clinical utility of this approach in diagnosing gastrointestinal disorders.

## Figures and Tables

**Table 1:** Summary of essential PCCT data acquisition and image reconstruction parameters (* the slice thickness of 3.0 mm was reformatted from the original 2.0 mm thickness in syngo.via).

Scanner Model		PCCT (NAEOTOM Alpha)		
Scan	Tube Voltage (kV)	120	140	Sn140
	Collimation (mm)	144 × 0.4		
	Rotation Time (s)	0.5		
	Pitch	0.6		
	CTDIvol (mGy)	Small/Medium/Large: 3/8/12 mGy		
Reconstruction	Image Type	Low-/High-Energy Images		
	Slice Thickness (mm)	3.0 *		
	Field of View (mm)	Small/Medium/Large: 220/350/420		
	Algorithm	Quantum Iterative Reconstruction (QIR-3)		
	Reconstruction Kernel	Qr40		

**Table 2:** Mass concentration measurements (mean ± standard deviation; units: mg/mL) and the range of quantification bias (measured mean mass concentration–nominal values) for both contrast materials.

Phantom Size	Nominal Concentration (mg/mL)	I (1.0) Bi (0.0)	I (2.0) Bi (0.0)	I (5.0) Bi (0.0)	I (0.0) Bi (0.6)	I (0.0) Bi (1.3)	I (0.0) Bi (2.5)	I (0.0) Bi (5.1)	Range of Bias
**Small**	120 kV	0.87 ± 0.94 0.24 ± 1.19	1.90 ± 0.96 0.11 ± 1.20	5.31 ± 0.87 −0.37 ± 1.08	−0.33 ± 0.84 0.87 ± 1.07	−0.26 ± 0.92 1.47 ± 1.16	−0.10 ± 0.79 2.58 ± 0.98	0.30 ± 0.83 4.74 ± 1.03	[−0.33, 0.31] [−0.37, 0.24]
	140 kV	0.99 ± 0.73 0.08 ± 0.87	1.89 ± 0.72 0.11 ± 0.83	5.12 ± 0.71 −0.13 ± 0.83	−0.32 ± 0.66 0.78 ± 0.78	−0.18 ± 0.72 1.31 ± 0.82	−0.06 ± 0.71 2.52 ± 0.81	0.25 ± 0.75 4.84 ± 0.88	[−0.32, 0.25] [−0.21, 0.15]
	Sn140 kV	0.75 ± 0.82 0.14 ± 0.64	1.78 ± 0.79 0.09 ± 0.63	5.05 ± 0.68 0.00 ± 0.57	−0.42 ± 0.91 0.69 ± 0.72	−0.28 ± 0.89 1.27 ± 0.70	−0.13 ± 0.82 2.52 ± 0.69	0.15 ± 0.76 5.00 ± 0.59	[−0.42, 0.15] [−0.05, 0.14]
**Medium**	120 kV	0.89 ± 1.18 0.05 ± 1.50	1.87 ± 1.22 0.10 ± 1.53	5.46 ± 1.12 −0.44 ± 1.49	−0.30 ± 1.22 0.92 ± 1.63	−0.16 ± 1.07 1.22 ± 1.45	−0.05 ± 1.12 2.41 ± 0.44	0.21 ± 1.15 4.91 ± 1.46	[−0.30, 0.46] [−0.44, 0.29]
	140 kV	0.94 ± 1.03 −0.04 ± 1.21	2.08 ± 1.04 −0.17 ± 1.15	5.33 ± 0.96 −0.21 ± 1.10	−0.25 ± 0.97 0.75 ± 1.18	0.04 ± 1.01 0.91 ± 1.21	0.01 ± 0.97 2.32 ± 1.11	0.19 ± 0.92 4.96 ± 1.06	[−0.25, 0.33] [−0.35, 0.12]
	Sn140 kV	0.76 ± 1.70 0.14 ± 1.25	1.77 ± 1.50 0.06 ± 1.04	5.17 ± 1.44 −0.10 ± 1.06	−0.26 ± 1.53 0.69 ± 1.12	−0.28 ± 1.64 1.15 ± 1.16	−0.04 ± 1.64 2.40 ± 1.21	0.49 ± 1.53 4.94 ± 1.08	[−0.28, 0.49] [−0.13, 0.14]
**Large**	120 kV	0.88 ± 1.08 0.44 ± 1.38	1.78 ± 1.16 0.01 ± 1.53	5.39 ± 1.17 −0.28 ± 1.50	0.05 ± 1.16 0.9 ± 1.48	−0.20 ± 1.21 1.33 ± 1.67	−0.19 ± 1.04 2.58 ± 1.31	−0.05 ± 1.08 5.20 ± 1.42	[−0.22, 0.39] [−0.34, 0.15]
	140 kV	0.76 ± 0.96 0.05 ± 1.16	1.49 ± 1.02 0.29 ± 1.22	5.13 ± 0.98 −0.08 ± 1.21	0.01 ± 0.95 0.30 ± 1.14	−0.01 ± 0.88 1.05 ± 1.11	−0.21 ± 0.92 2.54 ± 1.13	0.09 ± 0.93 5.04 ± 1.07	[−0.51, 0.13] [−0.33, 0.29]
	Sn140 kV	0.59 ± 1.84 0.22 ± 1.26	1.44 ± 1.67 0.18 ± 1.13	5.20 ± 1.86 −0.13 ± 1.28	−0.10 ± 1.76 0.40 ± 1.22	−0.32 ± 1.75 1.30 ± 1.20	−0.15 ± 1.75 2.48 ± 1.21	0.42 ± 2.04 4.93 ± 1.39	[−0.56, 0.42] [−0.24, 0.22]

## Data Availability

Data are contained within the article.
